# Influence of Bristle Stiffness of Manual Toothbrushes on Eroded and Sound Human Dentin – An *In Vitro* Study

**DOI:** 10.1371/journal.pone.0153250

**Published:** 2016-04-12

**Authors:** Mozhgan Bizhang, Katharina Riemer, Wolfgang H. Arnold, Julia Domin, Stefan Zimmer

**Affiliations:** 1 Department of Operative and Preventive Dentistry, Witten/Herdecke University, Witten, Germany; 2 Department of Biological and Material Sciences in Dentistry, Witten/Herdecke University, Witten, Germany; Glasgow University, UNITED KINGDOM

## Abstract

**Aim:**

The aim of this study was to determine the influence of manual toothbrushes with different bristle stiffness on the abrasivity on eroded and sound human dentin.

**Materials and Methods:**

Dentin specimens were made from impacted third molars and attributed to three groups: erosion-abrasion (EA), abrasion (A) and erosion (E). The specimens from EA and E were treated with 1% citric acid (pH 2.3) for 1 min rinsed, and neutralized with artificial saliva for 15 min. This cycle was repeated five times. Thereafter, specimens from EA and A were treated with three toothbrushes types with different bristle stiffness (soft, medium, and hard) in a custom-made toothbrushing machine. The brushing was performed at a load of 3 N with a toothpaste slurry for 630 s. This procedure was repeated five times, in group EA after each erosion cycle. EA and A groups passed through five cycles with a total of 6300 strokes. The abrasivity was analyzed by contact-free profilometry. Kruskal-Wallis and Mann-Whitney U tests were performed for statistical analysis.

**Results:**

With respect to bristle stiffness there was no statistically significant difference in dentin loss within the EA group. In group A, a statistically significantly higher dentin loss was found for the soft in comparison to the hard bristles. No statistically significant differences were measured between soft/medium and medium/hard toothbrushes. The amount of dentin loss from specimens in the EA group was significantly higher than in the A group.

**Conclusions:**

Within the limitations of this study, the dentin loss in the Abrasion group was higher with soft bristles than with hard ones. This result might have an influence on the toothbrush recommendations for patients with non-carious cervical lesions.

## Introduction

In a systematic review it was reported that the prevalence of tooth wear in adults was increasing from 3% at the age of 20 years to 17% at the age of 70 years [1]. Approximately 60% of people with dental erosions reported problems with dentin hypersensitivity, which is a possible side-effect of erosive enamel and dentin loss [2]. However, the etiology of tooth wear is multi-factorial and a result of the interaction of physical and chemical influences [3]. After softening of the dental hard tissue by erosive agents, mechanical factors such as abrasion may result in further loss of tooth surface [4].

Milosevic demonstrated the probable associations between erosion and toothbrushing habits [5]. For abrasion, most attention has focused on toothpaste, and dentin loss appears to correlate with its RDA value (Relative Dentin Abrasivity) [6]. In addition to the abrasivity of the toothpaste, the type of brush and the applied brushing force are known to be relevant factors for the loss of tooth surface [7]. For manual toothbrushes, Mierau and Spindler observed that individuals with multiple recessions had a mean brushing force of 3.75 N compared to 2.12 N when no recessions were present [8]. Other studies reported toothbrushing forces for manual toothbrushes ranging between 2.3 N and 3.23 N [9–13]. Furthermore, the filament diameter of the toothbrush has also been suggested as an important co-factor for abrasion [2, 14–16]. However, the literature is controversial regarding cleaning and abrasion with hard and soft bristles. In a randomized controlled trial, Zimmer et al. demonstrated better cleaning for hard filaments as compared to soft ones [17]. However, this better cleaning efficiency was associated with increased soft tissue abrasion. [17]. Wiegand et al. found that an experimental flat-trim toothbrush with a 0.2 mm filament diameter caused higher enamel loss after an erosive-abrasive challenge than those with 0.15 or 0.25 mm diameter [18]. In another clinical study, Versteeg et al. showed that the soft toothbrush “Sensodyne^®^ Sensitive” was more gingiva-abrasive than both the ADA toothbrush and the Oral-B^®^ Sensitive Advantage^®^. They concluded that gingival abrasion also depends on the brush-head designs [19].

Several methods have been introduced in dentistry to measure loss of hard tissue. Profilometry determines the spatial loss, whereas microradiography determines the total mineral content. Profilometry can provide a differential insight into the effects on mineralized and demineralized tissue [20].

Dentists often recommend soft bristles for patients with erosions or non-carious cervical lesions. The present study was designed to test which bristle stiffness is suitable for patients with gingival recessions resulting in dentin exposure. The aim of this study was to determine the abrasivity of three toothbrush types with different filaments on eroded and sound dentin. The primary hypothesis was that the use of a toothbrush with hard bristles would result in more hard tissue loss than soft bristles on both eroded and sound dentin. The secondary hypothesis was that the degree of abrasivity on eroded dentin would be more than that on sound dentin.

## Materials and Methods

### Dentin specimens

Based on a mean effect size of 1.2 (± 0.8), a power of 80% and a significance level of 5% (*p* < 0.05) the sample size was determined to be nine. The sample size calculation was performed with the G*Power software (version 3.0; http://www.psycho.uni-duesseldorf.de/abteilungen/aap/gpower3) [21]. The protocol for the collection of teeth for this in-vitro study was approved by the local ethics committee of the Witten/Herdecke University, Witten, Germany (No. 116/2013). On their first visit, the patients from the dental clinic of the Witten/Herdecke University could check the box saying “allow use of the extracted teeth for research”. Only the extracted teeth with prior written consent were collected. In addition, all patients were verbally informed that their molars would be used for research purposes. The teeth were transferred to the investigators anonymously, so that an identification of one individual tooth was impossible.

Sixty caries-free human third molars were gained and stored in a 0.1% thymol solution. From each tooth, one dentin specimen was obtained from the labial or lingual surface of the crown by using a trephine bur of 6 mm diameter (Hager Meisinger GmbH, Neuss, Germany). After removal of the enamel layer the dentin specimen was inspected under a dissecting microscope (10 x magnification).

**Fig 1:**
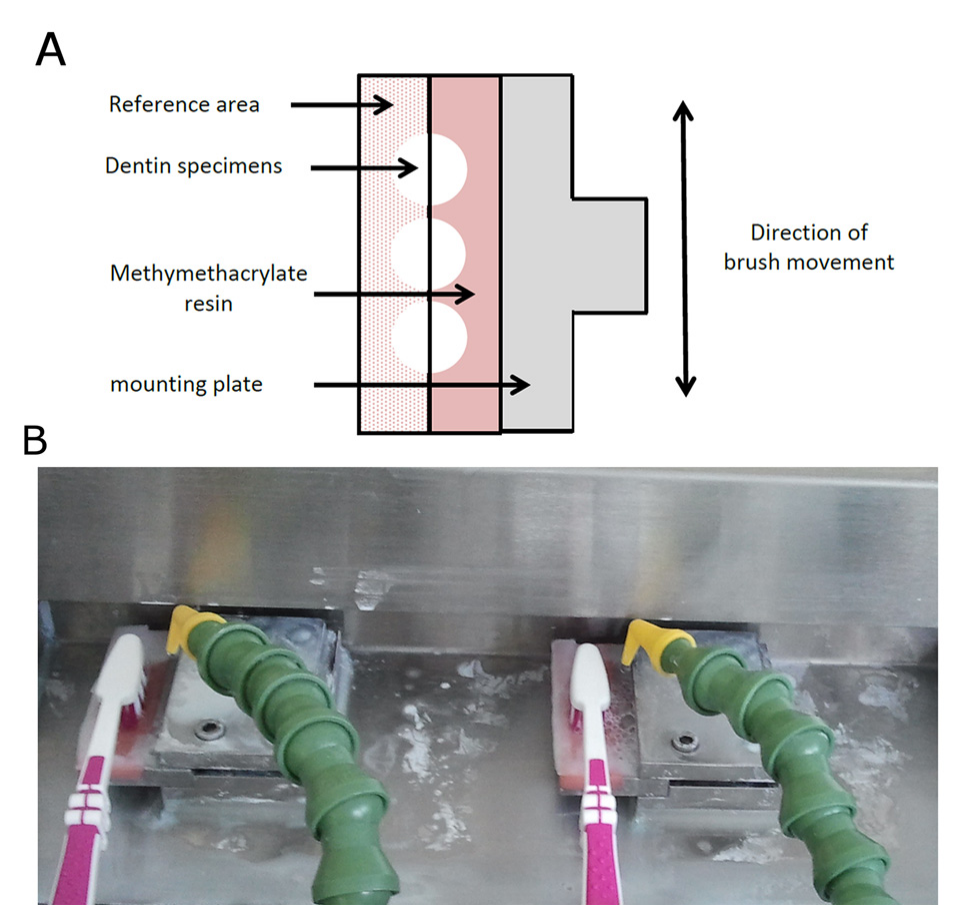
Dentin Specimens. (A) Schematic diagram of the embedment on the mounting plate.(B) The specimens were fixed in custom-made appliances in the brushing machine.

The surfaces of the dentin specimens (6 mm in diameter and 2 mm in height) were ground flat and polished with abrasive paper progressively up to 1000 grit on an automated polishing machine (EXAKT, Norderstedt, Germany). The dentin specimens were inserted into custom-made plates for the toothbrushing machine. Thereafter, one half of the dentin surface was covered with an adhesive tape (Tesa, Beiersdorf, Hamburg, Germany) in accordance with the direction of the brushing movement (Panel A and B of Fig 1)

### Experimental protocols

In order to rehydrate the specimens prior to the experiment, they were immersed in artificial saliva composition for 24 hours. Thereafter a randomized distribution of the specimens into the following three groups was carried out:

- Erosion-Abrasion group (EA, n = 27) (subgroups 1–3)
- Abrasion group (A, n = 27) (subgroups 4–6)
- Erosion group (E, n = 6) (subgroup 7)

### Erosion-Abrasion protocol

Twenty-seven specimens were randomly assigned to one of the three subgroups with different bristle stiffness, selected from Dr. Best High-Deep toothbrushes (GlaxoSmithKline, Bühl, Germany):

- Subgroup 1: hard, bristles with 0.23 mm diameter (n = 9)
- Subgroup 2: medium, bristles with 0.20 mm diameter (n = 9)
- Subgroup 3: soft, bristles with 0.18 mm diameter (n = 9)

**Fig 2:**
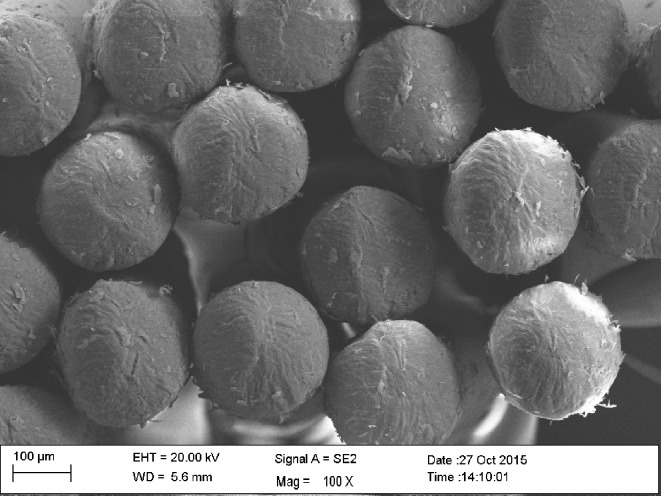
Dr. Best High-Deep, hard, at 300x and 117x magnification (white and color filaments).

**Fig 3:**
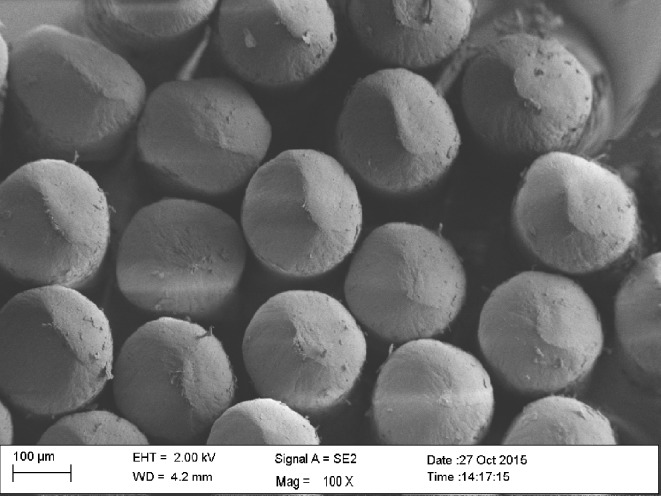
Dr. Best High-Deep, medium at 300x and 117x magnification (white and color filaments).

**Fig 4:**
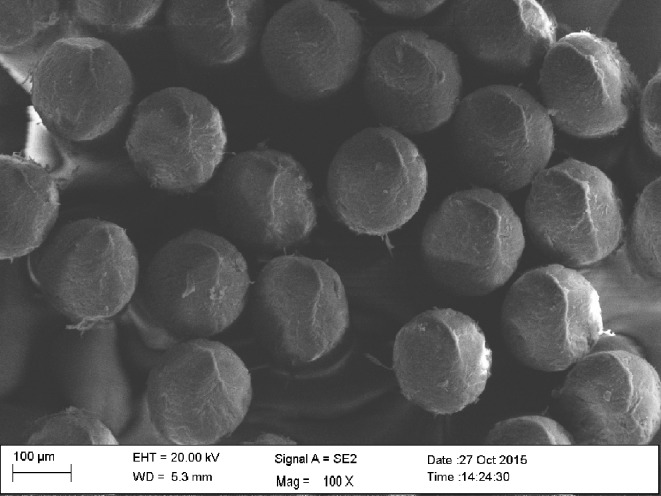
Dr. Best High-Deep, soft, at 300x and 117x magnification (white and color filaments).

All three Dr. Best High-Deep toothbrushes featured a flexible brush-head, a two-level bristle design, and end-rounded filaments. The toothbrush heads were of the same dimension and showed differences only in the diameters of the filaments and in the number of filaments per tuft. In order to evaluate end-rounding of the bristles and to count the bristles per tuft, scanning electron microscopy (SEM) was carried out with a Zeiss Sigma VP (Zeiss, Oberkochen, Germany). For this purpose, the toothbrush heads were sputtered with gold-palladium and mounted on standard specimen-holders. Photomicrographs were taken at an acceleration voltage of 2 kV with the secondary electron detector (Figs 2, 3 and 4). The numbers of bristles/tuft were 30 to 32 for hard, 38 to 44 for medium, and 46 to 52 for soft. The length of bristles was similar (length, 9.2–10.5 mm) for all the toothbrushes.

**Fig 5:**
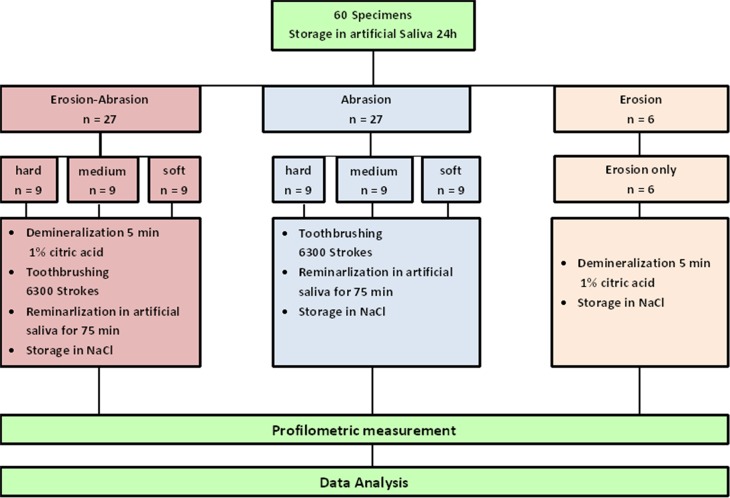
Study design.

Before they were brushed in the toothbrushing machine, the respective specimens (group EA) were demineralized for 60 seconds, than remineralize for 15 min., brushed for 630 s, and again remineralized for 15 min. This cycle was repeated five times for group EA. Citric acid solution (1%, pH 2.3) was used for 60 s at room temperature for the demineralization process. The pH-value and the total time of demineralization were chosen as employed in other studies [20, 22]. The specimens were brushed with the automatic brushing machine at a load of 3N with 1260 linear strokes within the 630 s of the treatment [23] Toothpaste and artificial saliva at a ratio of 1:3 were used for brushing according to EN ISO 11609:2010 standard (Dentistry-Toothpastes: Requirements, test methods and marking). The second cycle started after the second remineralization. (Fig 5)

The custom-made tooth brushing machine (“UWH-DentTest”, Ingpuls, Bochum and Department of Operative and Preventive Dentistry of the Witten/Herdecke University) consists of six test devices for diverse combinations of toothbrushes and artificial tooth models. The artificial tooth models are fixed on attachments in the test chamber. The data is collected with a connected PC. Linear brushing movements are generated by the machine by a linear forward and backward-stroke at the same level. The flow rate of the slurry was set to 10 ml/minute, with each specimen receiving new slurry every two minutes. Tap water was used in the intermediate washing cycle for 30 s. All these parameters were set-up and monitored by the control device of the test station. The artificial saliva used in the study was prepared according to Klimek et al. [24]. The toothpaste applied was Eurodont toothwhite (Dr. Scheller Durodont GmbH, Eislingen, Germany) with an RDA value of 80. This toothpaste is one of the most commonly used in Germany and is recommended for daily use. After five brushing cycles, the specimens were stored in Ringer’s solution (0.9% NaCl, Deltaselect GmbH, Pfullingen, Germany) before being analyzed. The total of 6300 brushing strokes were calculated to be equivalent to 21 months of brushing, based on a brushing time of 120 s twice-daily brushing for all teeth [25]. Based on this assumption, the maximum contact time for one tooth-surface per day is 5 s [6].

### Abrasion protocol

Twenty-seven specimens were randomly assigned to one of the three subgroups as in the EA group. They were then brushed as in the EA protocol, but without the erosion cycles (Fig 5):

- Group 4: hard (n = 9)
- Group 5: medium (n = 9)
- Group 6: soft (n = 9)

### Erosion protocol

Six specimens were exposed to 60 seconds demineralization in citric acid (1%, pH 2.3) solution at room temperature. After they were rinsed with deionized water, the erosive challenge was repeated five times (subgroup 7). These samples served as control to determine the degree of erosion and were not subjected to toothbrushing. (Fig 5)

### Measurement of dentin loss

**Fig 6:**
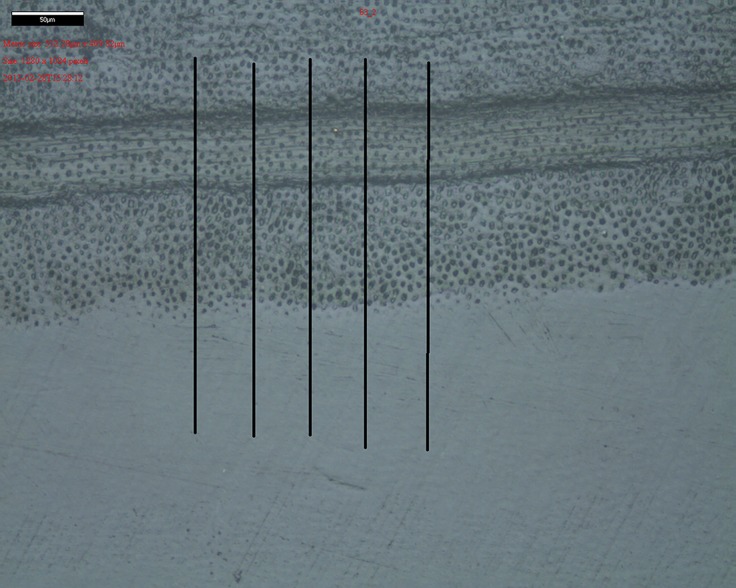
Measurements of step height.

The adhesive tape was removed to expose the reference areas. To avoid dehydration, the specimens were air-dried just before the measurements. Immediately after the profilometry measurement, the specimens were again stored in Ringer’s solution. The dentin loss was evaluated by optical profilometry (InfiniteFocus G3, Alicona, Raaba, Austria) with its corresponding software (IFM 2.2). Dentin loss was determined in relation to the reference surfaces using a 3D image, taken perpendicular to the brushing movement with standardized magnification and resolution. All measurements were made under constant moisture control. Before each measurement, the sample surface was covered with distilled water for 30 s. The water was removed with absorbent tissue without contact to the specimen surface. Surface scans were performed with a 20 mm objective and a vertical resolution of 150 nm. The scans resulted in a total scanned area of 717 x 500 μm of which one half was covered with tape and the other half was exposed to the toothbrush. Five profiles were gathered for each specimen, one at the midpoint of the plate and two profiles at 50 μm and 100 μm right and left of the midpoint, respectively. Five lines with a length of 300 μm each and a distance of 50 μm from each other were drawn within this chosen area to verify the corresponding dentin loss (Fig 6). The mean of five measurements of the step height between the areas was the primary outcome of this study. The specimens were fixed in custom-made appliances allowing exact repositioning of the specimens in both the brushing machine and the Profilometer.

### Data analysis

IBM SPSS Statistics 20 software was used for statistical analysis. The Kolmogorov-Smirnov Test showed an inhomogeneous distribution of the data. Owing to the asymmetric data distribution of dentin loss, we applied non-parametric tests for statistical analysis. Consequently, Kruskal−Wallis test was used for global testing and Mann−Whitney U-test for head-to-head comparisons. The significance level was set to *p* ≤ 0.05. Alpha was adjusted to avoid alpha-error accumulation with regard to multiple paired comparisons. Therefore, *p* < 0.02 was calculated for primary outcomes and for each segment.

## Results

**Fig 7:**
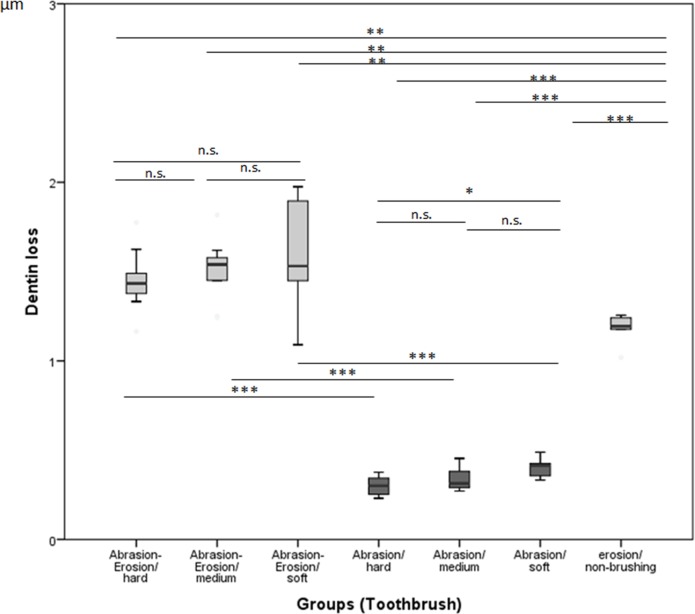
Dentin loss (μm) for different toothbrush types within the AE, A, and E groups. Horizontal bars are indicating statistically significant differences within groups (EA, A) for hard, medium, and soft bristles, * p < 0.02, Mann-Whitney U-test) as well as between groups (n.s., not significant; *p < 0.05; ** p < 0.01; ***p < 0.001, Mann-Whitney U-test).

In the A group, the soft-bristle toothbrush [0.41 (0.35–0.44 μm)] led to higher dentin loss than the hard-bristle toothbrush [0.30 (0.25–0.35 μm)] (Kruskal-Wallis test and Mann-Whitney U-test, p< 0.02). No difference was measured between toothbrushes with soft and medium bristles [0.31 (0.29–0.42 μm)] and those with medium and hard bristles (Kruskal-Wallis test and Mann-Whitney U-test, p< 0.02). EA group showed no statistically significant differences with respect to the bristle stiffness: soft [1.53 (1.44–1.91) μm)], medium [1.54 (1.35–1.59 μm)], and hard [1.43 (1.35–1.56 μm)] (Kruskal-Wallis test, p< 0.02). The E group showed an average dentin loss of 1.19 μm [1.14–1.24 μm)] (Table 1 & Fig 7).

**Table 1:** Median (25th and 75th percentiles) Dentin loss after 6300 strokes (μm).

	Median (μm)	25th percentile (μm)	75th percentile (μm)
**Erosion-Abrasion**			
Hard	1.43	1.35	1.56
Medium	1.54	1.35	1.59
Soft	1.53	1.44	1.91
**Abrasion**			
Hard	0.30	0.25	0.35
Medium	0.31	0.29	0.42
Soft	0.41	0.35	0.44
**Erosion**			
Erosion	1.19	1.14	1.24

The amount of dentin loss from the eroded dentin specimens (EA group) was significantly higher for all toothbrush types when compared with that from specimens of the A group (Mann- Whitney U test, p<0.05). Table 1 shows the medians and 25^th^ and 75^th^ percentiles of the data.

Dentin loss in the E group was significantly higher in comparison to the A group but less in comparison to the EA group (Kruskal-Wallis test and Mann- Whitney U-test, p< 0.05)

## Discussion

This study investigated the influence of toothbrushes with different bristle stiffness on eroded and sound dentin specimens. A relationship between bristle stiffness (0.18–0.23 mm diameter) and dentin loss was found in the A group. In the EA group, no statistically significant differences were found between bristle stiffness and dentin loss. The primary hypothesis was rejected.

**Fig 8:**
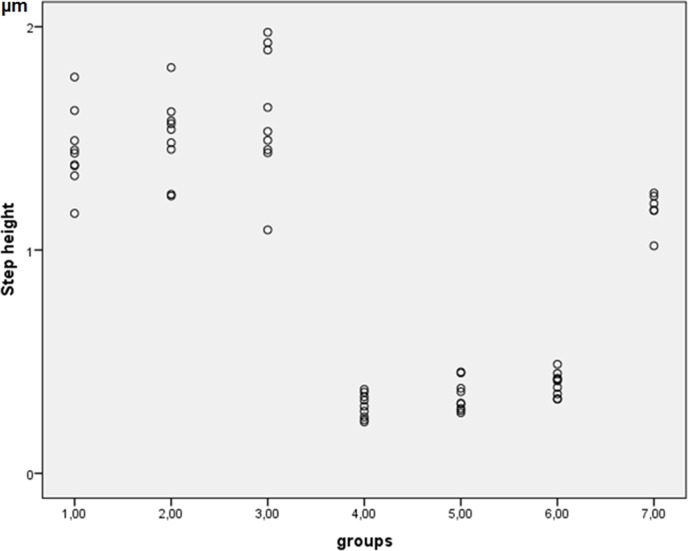
Scatterplot of the relationship between step height and data on erosion and abrasion.

In contrast to our results, which showed large standard deviations (Fig 8), Wiegand et al. [26] found, that the abrasion of eroded dentin was reduced with an increase in the filament diameter of the toothbrush.

The abrasion of eroded dentin was significantly higher than of sound dentin for each toothbrush type (soft, medium, and hard). This result is in accordance with findings for power toothbrushes [27].

In-vitro studies to examine dental erosion have both advantages and disadvantages. Patient-related parameters such as compliance, saliva, oral hygiene, cleaning force, and behavioral factors can be eliminated in standardized in-vitro studies, but the use of artificial saliva and extracted, non-erupted teeth cannot exactly reflect the conditions in vivo. For a verification of abrasivity of different oral hygiene products, in-vitro studies represent a standardized method, giving no absolute values but allowing for a comparison of the examined product groups [15, 22, 28]. Nevertheless, in-vitro studies offer less evidence than clinical trials.

The results of this study demonstrated that soft bristles (0.18 mm diameter) caused to more dentin loss than the medium (0.2 mm diameter) or the hard (0.23 mm diameter) bristles. These results confirm those of two other studies [15, 28]. The toothbrushes described by Wiegand et al. [27] were flat trim, while those in the present study were high-deep in design. In contrast, other authors have demonstrated that bristle stiffness does not play an important role in abrasiveness [29–31]. The soft toothbrush is thinner and more flexible. The higher abrasivity of soft toothbrushes is probably caused by the more homogenous, greater flexion of filaments and smooth dispersion of toothpaste on the dental hard-tissue surface, as well as by the larger surface area. Therefore, more toothpaste can adhere to the dental surface and cause higher abrasivity. The number of filaments per tuft also contributes to the hardness of a toothbrush. In the present study, the numbers of filaments/tuft differed. The soft brush had more filaments than the other brushes. Robertson and Wade reported that the total number of filaments in a brush was less important than the filament diameter in the effective removal of plaque [32]. Hunter and West stated that various individual factors, such as toothbrushing method, bristle stiffness, cleaning force, cleaning time, and frequency, are involved in dental hard tissue loss [33]. Primary criteria for the selection of toothbrushes in this study were the presence of uniform parameters e.g., design, form, and size, differing only in filament diameters. Artificial saliva with mucin was mixed according to EN ISO 11609:2010 standard (Dentistry-Toothpastes: Requirements, test methods and marking) for the slurry.

Furthermore, this study was carried out with similar brushing techniques and forces. A brushing force of 3 N (300 g) and a linear back-and-forth movement were selected for this study [22, 34]. Various brushing forces and techniques can be found in the literature [35] and might be due to differences in the selection of study groups or to methodological variance. However, no systematic review of brushing force or brushing technique has thus far been performed. It has also been found that individuals with wedge-shaped lesions had significantly higher mean brushing force than those without cervical wear (2.9 ± 0.4 vs 2.1 ± 0.3 N) [36]. A 3 N brushing force and scrubbing movement might be of relevance to trauma in hard tissues.

In accordance with other studies [37–39] dentin specimens were used in the present study since dentin is known to be more affected by abrasivity of toothpastes than enamel. In addition dentists often recommend soft bristles for patients with erosions or with non-carious cervical lesions and it should be investigated in the present study whether this recommendation is correct. Profilometry was employed as several studies have used the same method for the determination of hard tissue surface loss [22, 40–42]. Although the presence of a smear and an organic layer [20, 43] have been found to be weaknesses of this method, Ganss et al. concluded in their study, that profilometry can provide a differential insight into the effects on mineralized as well as demineralized tissue [20].

One in situ study showed that soft and hard toothbrushes presented no significant differences in toothbrush abrasion of softened human enamel [44]. This result was based on the enamel surface and hence cannot be compared with the present data. Limitations of this study were the low number of specimens in the Erosion-Abrasion group and the in vitro setting with standardized parameters for brushing load and technique. This might not reflect the real circumstances in vivo. Another limitation is the restriction to one toothbrush type which, on the other hand, was necessary to study the influence of the bristle stiffness only.

## Conclusions

Within the limitations of this study, the following conclusions can be drawn:

- Linear brushing with 6300 strokes and a soft toothbrush under the conditions of 3 N brushing force and toothpaste slurry (RDA 80) on sound dentin results in higher abrasivity than brushing with medium or hard toothbrushes with high-deep design brush heads. The eroded dentin showed no statistically significant differences with respect to different bristle stiffness. No significant differences of abrasivity were found between soft, medium and hard toothbrushes on eroded dentin.
- The degree of abrasivity on eroded dentin for soft, medium and hard toothbrushes was more than that on sound dentin

However, further investigations for the eroded dentin group must be considered using a higher number of specimens.

## Supporting Information

S1 File Study data-file. (XLSX)
